# Examining firearm-related deaths in Mexico, 2015–2022

**DOI:** 10.1186/s40621-024-00519-z

**Published:** 2024-07-29

**Authors:** Eugenio Weigend Vargas, Michelle Degli Esposti, Stephen Hargarten, Laura Vargas, Jason E. Goldstick

**Affiliations:** 1 Institute for Firearm Injury Prevention, University of Michigan, 1109 Geddes Ave, Ann Arbor, MI 48109 USA; 2 Emergency Medicine, Medical College of Wisconsin, Milwaukee, WI USA; 3 Comprehensive Injury Center, Medical College of Wisconsin, Milwaukee, WI USA; 4 Department of Psychiatry, University of Colorado School of Medicine, Anschutz Medical Center, Aurora, CO USA; 5 Injury Prevention Center, University of Michigan, Ann Arbor, MI USA; 6 Department of Emergency Medicine, University of Michigan, North Campus Research Complex, Ann Arbor, MI USA; 7 Department of Health Behavior and Health Education, School of Public Health University of Michigan, Ann Arbor, MI USA

**Keywords:** Mexico/epidemiology, Firearm violence, Homicides, Suicides

## Abstract

**Background:**

Globally, Mexico is one of six countries with the highest level of firearm mortality. While previous studies have examined firearm mortality in Mexico before 2015, increases in violence since then highlight the need for an updated analysis. In this study, we examined changes in firearm-related deaths in Mexico from 2015 to 2022 and described these deaths by key demographic groups, incident location, and state of occurrence. Data came from Mexico’s *Instituto Nacional de Estadistica y Geografia* (INEGI), a federal agency that collects and reports national population data. We used descriptive statistics to analyze rates, proportions, and percentage changes in firearm mortality, and we displayed temporal trends using time plots and special trends using maps.

**Results:**

Firearm deaths increased in Mexico from 2015 to 2018 but slightly decreased from 2018 to 2022. Homicides presented the highest increase and the highest proportion of firearm-related deaths from 2015 to 2022. Victims were primarily males but rates among women increased at a higher proportion (99.5% vs 53.5%). One third of victims were 20–29y but rates among children and adolescents (10–9y) increased at a higher proportion. Most firearm-related deaths occurred in streets or public spaces but the percentage of incidents occurring in households have increased. State-level rates and percentage changes varied significantly. States with higher rates of firearm mortality coincide with those involving conflict among organized criminal organizations.

**Conclusion:**

Firearm mortality in Mexico is a major public health burden. The epidemiology of firearm-related deaths in Mexico varies by intent, demographics, location, and states. To mitigate this challenge, multiple solutions are required.

## Introduction

Previous studies have documented increases in firearm mortality in Mexico from 1990 to 2015 (Dare et al. 2019). Reports from nonprofit organizations and news outlets indicate further increases since 2015, (particularly firearm homicides associated with organized crime) (Calderon et al. 2020), but comprehensive characterization of those trends is lacking. Organized criminal groups continue to operate in Mexico and roughly 213 k firearms are trafficked from the US every year (McDougal et al. 2014). In this regard, further increases in firearm mortality would threaten the future economy and health of Mexico (Peters et al. 2020; Aburto et al. 2016), and yet the lack of precise epidemiological information limits the ability to address this growing national problem with evidence-based programs and policies. In this analysis, we document changes in firearm-related deaths in Mexico from 2015 to 2022 and describe these deaths by key demographic groups (e.g., sex, age, and urbanicity), incident location (e.g., households or public spaces) and states where they occurred.

## Methods

Data on causes of death were collected from Mexico’s *Instituto Nacional de Estadistica y Geografia* (INEGI), a national vital statistic database that has previously been used to examine firearm mortality in Mexico (Dare et al. 2019; Instituto Nacional de Estadística y Geografía 2023a). INEGI collects annual mortality data and provides information on year of occurrence (Instituto Nacional de Estadística y Geografía 2023b). We merged datasets from 2015 to 2022. We excluded deaths that occurred/registered before 2015 and those where year of occurrence was unknown (n = 432), as well as deaths that occurred outside of Mexico (n = 2).

In line with previous studies (Degli Esposti et al. 2023; Cunningham et al. 2018), we identified firearm deaths using the International Classification of Disease (ICD-10) codes for firearm homicide (X93–X95 and U01.4), firearm suicide (X72–X74), unintentional firearm deaths (W32–W34), and firearm deaths of undetermined intent (Y22–Y24). Firearm deaths were further disaggregated by sex and age group (< 10y; 10–19y; 20–29y; 30–39; 40–49y; 50–59y; 60–69y; 70y+) and geographic information (urbanicity, incident location, state of occurrence). Urbanicity was defined using INEGI’s predetermined categories of urbanicity (urban and rural). Similarly, incident location was defined using INEGI’s eight predetermined categories of where deaths occurred (household, school or office, sport fields, streets or public spaces, commercial areas, industrial areas, farms/ranches, and other).

We used descriptive statistics to examine pooled 2015–2022 rates, annual rates for 2015 and 2022 separately, as well as percentage changes (in rates) from 2015 to 2022 across categories of intent, sex, age groups, and state of occurrence. To obtain rates, we used population estimates (by year, sex, age group, and state) provided by Mexico’s *Consejo Nacional de Población* (CONAPO) (Consejo Nacional de Población 2024). We also examined the percentage of firearm related deaths within categories defined by urbanicity, location, intent, and demographics. We displayed these percentages for 2015, 2022, and the total pooled 2015–2022 period.

## Results

**Fig. 1:**
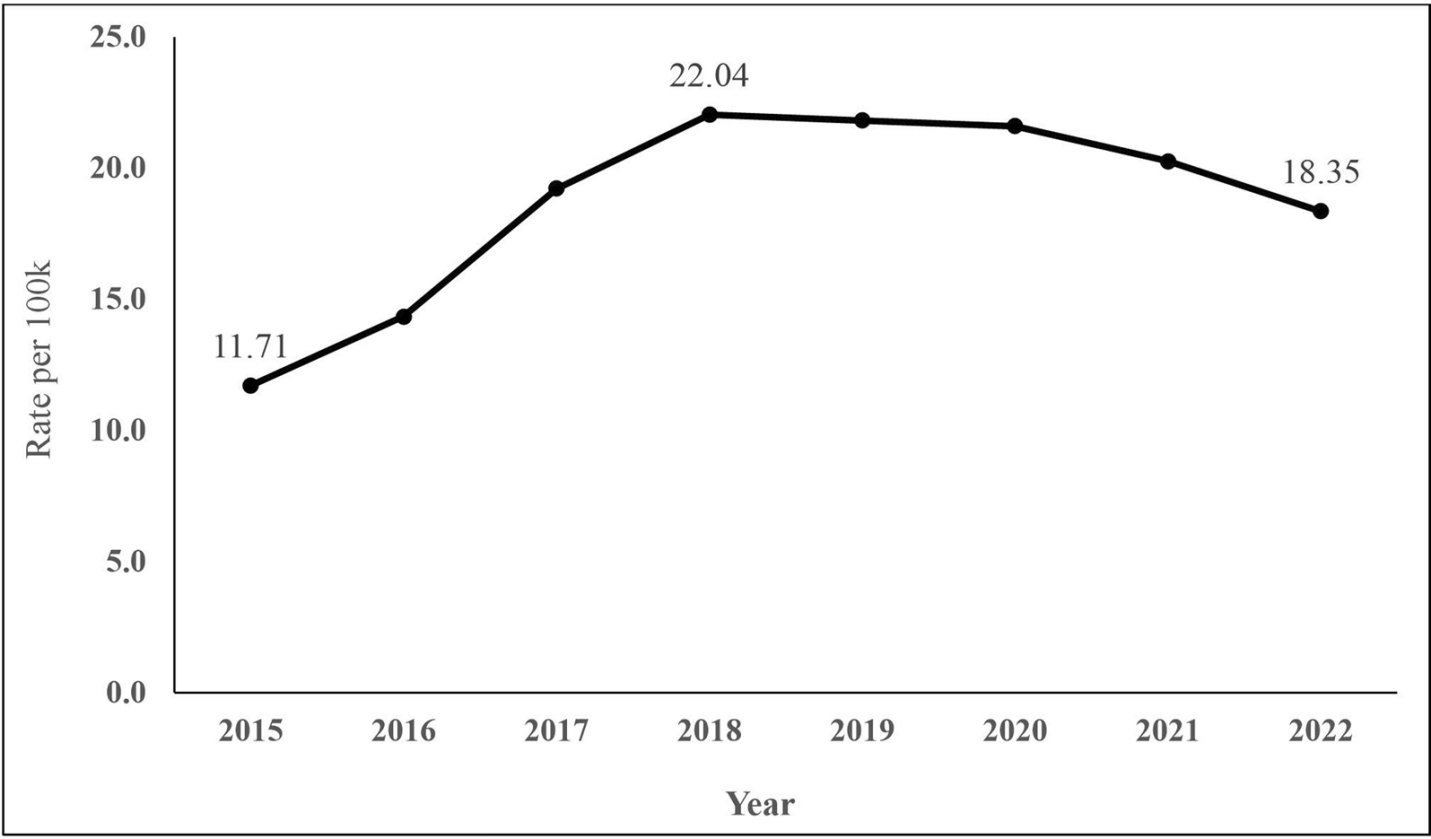
Rate of firearm mortality in Mexico, 2015–2022

We examined 188,397 firearm-related deaths in Mexico from 2015 to 2022. Rates of firearm-related deaths increased by 88.2% from 2015 to 2018 and decreased by 16.7% from 2018 to 2022 (Fig. 1). Homicide accounted for 92.2% of firearm deaths (Table 1) during this period and experienced the highest percentage increase (62.7%) from 2015 (10.37 per 100 k) to 2022 (16.87 per 100 k). Rates of undetermined firearm-related deaths and unintentional shootings also increased during this period (Table 1), while firearm suicide decreased by 23.4% (from 0.47 per 100 k in 2015 to 0.36 per 100 k in 2022).

**Table 1:** Rates of firearm deaths, percentage increases in rates, and the proportion of firearm deaths by intent, sex, and age groups, 2015–2022

	Rates per 100 k			Percentage	
	2015	2022	2015–2022	Rate change 2022 versus 2015	Proportion of all firearm deaths 2015–2022
Total	11.71	18.35	18.71	56.70	100.00
Intent					
Homicide	10.37	16.87	17.25	62.68	92.19
Suicide	0.47	0.36	0.41	− 23.40	2.17
Unintentional shootings	0.50	0.57	0.52	14.00	2.79
Undetermined	0.37	0.55	0.53	48.64	2.85
Sex					
Males	21.95	33.64	34.77	53.26	91.17
Females	1.83	3.65	3.23	99.45	8.83
Age groups (years)					
0–9	0.29	0.31	0.36	6.90	0.35
10–19	5.16	8.65	8.08	67.64	8.12
20–29	19.72	31.84	33.04	61.46	31.88
30–39	22.45	33.82	35.66	50.65	28.62
40–49	17.69	27.43	27.41	55.06	18.76
50–59	10.92	15.42	15.46	41.21	8.09
60–69	7.36	8.15	8.92	10.73	2.90
70+	4.39	4.62	4.78	5.24	1.28

There were few observations where sex (< 1%) and age (< 4%) were missing. Hence, the proportion of firearm deaths among these categories (5th column) are based on specified data (excluding missing data). Percentage changes (4th column) are based on rates rounded to the hundredth place

Males represented 91.2% of firearm-related deaths (Table 1). However, increases from 2015 to 2022 were larger among women than men (99.5% vs 53.3%). Sex of decedent was missing in < 1% of deaths. Individuals ages 20–29y represented close to one third of firearm-related victims (31.9%) but rates were higher among individuals ages 30–39y (35.66 per 100 k). Across age groups, the largest percentage rate increase (67.6%) from 2015 to 2022 was among those aged 10–19y (from 5.16 to 8.65 per 100 k). Age of decedent was missing in 3.7% of deaths.

The percent of firearm deaths occurring in urban areas increased from 76.8% in 2015 to 86.8% in 2022 (Table 2). Across all intents, most deaths occurred in urban areas (Table 2). However, roughly one out of every four firearm suicides (27.2%) occurred in rural areas, the highest proportion across all intents. By age group, 23.1% of firearm deaths among those ages 0 to 9 and over 25.0% of those 60–69y as well as 70+ years of age occurred in rural areas (Table 3). Urbanicity was missing in 8.4% of deaths.

**Table 2:** Percentage of firearm deaths (across years, intent, and sex) by urbanicity and location, 2015–2022

	Year			Intent				Sex	
	2015–2022 (%)	2015 (%)	2022 (%)	Firearm homicides (%)	Firearm suicides (%)	Unintentional shootings (%)	Undetermined intent (%)	Males (%)	Females (%)
Urbanicity									
Urban	84.07	76.82	86.80	84.40	72.77	80.22	86.36	83.83	86.49
Rural	15.93	23.18	13.20	15.60	27.23	19.78	13.64	16.17	13.51
Location									
Households	14.96	13.97	17.69	13.17	73.31	26.85	20.56	13.82	26.97
Schools or office	0.26	0.35	0.22	0.24	0.80	0.64	0.39	0.25	0.36
Sporting areas	0.30	0.31	0.38	0.30	0.21	0.37	0.27	0.30	0.21
Street or public spaces	67.33	66.86	64.76	69.20	10.31	49.95	60.33	68.42	55.89
Commercial areas	3.42	3.22	3.65	3.51	1.82	0.96	2.65	3.38	3.88
Industrial areas	0.47	0.53	0.49	0.48	0.51	0.27	0.18	0.50	0.15
Farms or ranches	3.02	4.66	1.99	2.95	4.68	4.80	2.83	3.08	2.27
Other	10.25	10.09	10.82	10.15	8.36	16.15	12.78	10.24	10.26

Observations for urbanicity (8.4%) and for location (17.3%) were missing. To estimate percentages for each category, missing data was excluded

**Table 3:** Percentage of firearm deaths (across age groups) by urbanicity and location, 2015–2022

	0–9 (%)	10–19 (%)	20–29 (%)	30–39 (%)	40–49 (%)	50–59 (%)	60–69 (%)	70 + (%)
Urbanicity								
Urban	76.92	84.15	86.01	85.42	83.05	80.00	74.74	70.76
Rural	23.08	15.85	13.99	14.58	16.95	20.00	25.26	29.24
Location								
Households	35.48	14.54	11.56	13.73	16.61	21.77	28.18	49.22
Schools or office	0.38	0.31	0.18	0.25	0.37	0.42	0.16	0.40
Sporting areas	0.00	0.42	0.33	0.29	0.28	0.22	0.22	0.15
Street or public spaces	44.02	68.39	71.61	69.06	64.95	58.82	51.61	31.78
Commercial areas	2.85	2.58	3.04	3.67	4.13	4.15	3.85	2.27
Industrial areas	0.38	0.32	0.37	0.49	0.58	0.68	0.74	0.66
Farms or ranches	3.04	2.83	2.55	2.54	2.96	4.33	6.07	6.57
Other	13.85	10.60	10.35	9.98	10.12	9.62	9.18	8.94

To estimate percentages for each category, undetermined/missing data was excluded

When examining incident location (Table 2), 67.3% of deaths occurred in the street or in public spaces. However, the percentage of incidents occurring in households increased from 14.0% in 2015 to 17.7% in 2022. While homicides occurred mainly in streets and public spaces (69.2%), firearm suicides occurred primarily in households (73.3%). The proportion of firearm-related deaths among women that occurred in households (27.0%) is larger than the percentage among men (13.8%). More than 64.0% of firearm-related deaths among those age 10–19y, 20–29y, 30–39y, and 40–49y occurred in streets or public spaces (Table 3), while the proportion of incidents occurring in households was higher among victims 0–9 years of age (35.5%) and among those older than 69 years of age (49.2%). Incident location was missing in 17.3% of deaths.

**Fig. 2:**
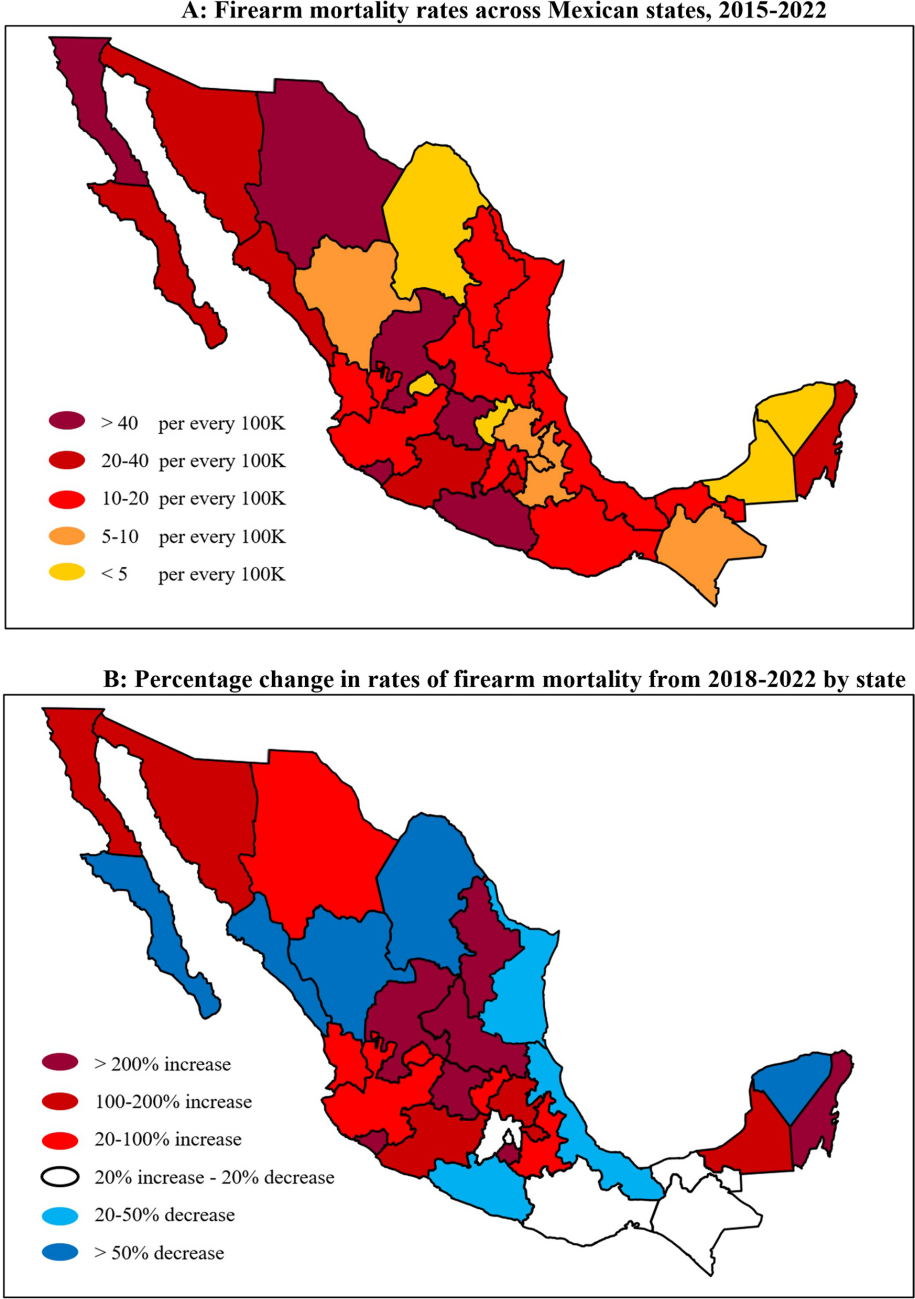
**A** Firearm mortality rates across Mexican states, 2015–2022, **B** Percentage change in rates of firearm mortality from 2018 to 2022 by state. The state where the death occurred was missing in < 2% of incidents. Percentage changes in the state of Yucatan are based on counts of < 30. Interpret with caution

The five states with the highest rates of firearm mortality per 100 k from 2015 to 2022 (Fig. 2A) were Colima (66.91), Baja California (48.23), Zacatecas (44.35), Chihuahua (43.51), and Guanajuato (42.87). The five states with the lowest rates of firearm mortality per 100 k were Yucatan (0.83), Aguascalientes (3.45), Coahuila (4.05), Queretaro (4.97), and Campeche (4.98). Most states experienced significant shifts in rates of firearm mortality from 2015 to 2022, however, these changes were not homogenous across states (Fig. 2B). For example, five states experienced a decline of more than 50.0% (e.g., Baja California Sur decreased by 84.7%) while seven states had increases of more than 200.0% (Quintana Roo increased by 435.6%). State of occurrence was missing in 1.6% of deaths.

## Discussion

Firearms account for a growing burden of avoidable deaths in Mexico, with rates increasing substantially from 2015 to 2022. In contrast to the US where firearm suicides represented 56.1% of firearm-related deaths in 2022 (Centers for Disease Control and Prevention 2024), homicides are the biggest challenge for reducing firearm-mortality in Mexico, representing 92.2% of firearm deaths from 2015 to 2022. In this regard, conflicts involving organized criminal groups play an important role (Calderon et al. 2021). Our results on the geography of firearm-mortality in Mexico suggests that these deaths are more likely to occur in states involving conflicts among organized criminal groups as reported by the Congressional Research Service in 2022 (Mexico 2024). Additionally, our results indicate that rates of firearm-deaths varied over time. This suggests the need to rely on the most recent epidemiological data to dictate public health priorities and response activities.

Our results suggest demographic- and location-specific challenges around firearm mortality that require complementary approaches. Rates among women have increased at a higher proportion than men, coinciding with increases in femicides in Mexico in recent years (Hernandez Gress et al. 2023). While firearm-related deaths among women occurred more frequently in the street/public spaces, our results indicate that one out of four firearm-related deaths among women occur in a household, suggesting homicides related to domestic violence. Similarly, individuals aged 10–19y presented the highest relative rate increase from 2015 to 2022. As firearms have become a major cause of death for children and adolescents in Mexico (Castilla Peon et al. 2023), focusing on curbing this trend is critical. Also, as 35.5% of firearm deaths among children (0 to 9) occur in households, it is important to focus on reducing access to firearms among children as well as reducing firearm availability where there is domestic violence. Moreover, while rates of firearm suicides and unintentional shootings are not as high as firearm homicides, they occur more frequently within households. At the same time, close to 27.2% of firearm suicides occur in rural areas despite such areas representing 21.0% of the population (Instituto Nacional de Estadística y Geografía 2024). Policymakers could examine and potentially replicate US policies to limit access to firearm for domestic abusers, children, adolescents, and those at risk of suicide. Suicide prevention policies could include the adoption of extreme risk protection orders, a policy that has been associated with a reduction in firearm suicides in US states (Kivisto and Lee 2018). Moreover, while firearm laws are regulated at a federal level, future studies could evaluate the adequate implementation of Mexico’s firearm laws.

In addition, while firearm mortality increases were seen in official data following the start of the COVID-19 pandemic period (2020–2022) in the US (Sun et al. 2022), this did not occurred in Mexico. Rates in Mexico presented decreases of 1.0% from 2019 to 2020 and 6.2% from 2020 to 2021. These changes followed a similar decrease of 1.0% from 2018 to 2019 (pre pandemic). Nonetheless, within categories of intent, trends in rates of death by unintentional shootings increased 50% from 2019 to 2020 (0.44 to 0.66 per 100 K) and maintained similar levels during 2021 (0.60 per 100 k). Future studies should examine associations between factors arising from the COVID-19 pandemic and firearm mortality.

Findings from this analysis have implications beyond Mexico. US federal agencies have reported that many firearms recovered in Mexico and traced by the Bureau of Alcohol Tobacco Firearm and Explosives (ATF) originate in the US (Bureau of Alcohol Tobacco Firearms and Explosives (ATF) 2022). Researchers have also reported a strong association between US firearm markets (manufacturing and imports) and rates of firearm homicides in Mexico (Perez Esparza et al. 2020; Weigend Vargas et al. 2024). As studies have documented increases in firearm-mortality in the US after 2014 and have reported firearms as the number one cause of death among children and adolescents (Goldstick et al. 2022), efforts to address these challenges should also consider firearm mortality in countries that are major recipients of legal and illegal US firearms. Additionally, bilateral agreements between Mexico and the US to reduce firearms trafficking and increase oversight of firearm exports should be considered.

There are limitations to our study. Our analysis is based on mortality that has been reported to INEGI. However, as presented in this study, there is missing data across numerous categories, including information on state and year of occurrence as well as incident location. This highlights the need for more comprehensive and timely data collection. Potentially, Mexico could replicate US efforts in creating the National Violent Death Reporting System (NVDRS) (Centers for Disease Control and Prevention 2024), starting with pilot programs in selected states or cities. Despite these limitations, INEGI is recognized as a reliable source in reporting mortality in Mexico and has been used in previous studies examining firearm mortality (Castilla Peon et al. 2023). Another limitation is that we are not characterizing increases in nonfatal shootings. As studies have documented that nonfatal shootings in Mexico are more likely to occur than fatal shootings (Weigend Vargas and Perez 2022), future research should focus on conducting epidemiological studies to describe this challenge. Finally, state rates are based on resident population, which may not perfectly proxy the susceptible population within a given year.

Firearm mortality in Mexico is a major public health burden. The epidemiology of firearm-related deaths in Mexico varies by intent, demographics, and states. To mitigate this challenge, multiple solutions are required. These include programs and policies to mitigate violence associated with organized criminal groups, including efforts to reduce firearms trafficking from the US. Federal and state policies should also consider complementary public health approaches, including better data compilation and the potential adaptation of policies and programs that have been effective at reducing firearm mortality in other countries.

## Data Availability

Data used in this research is publicly available through Mexico’s *Instituto Nacional de Estadistica y Greografia* (INEGI). Data can be downloaded from this page https://www.inegi.org.mx/programas/mortalidad/#microdatos. Data compiled by the authors is available upon reasonable requests.

## Abbreviations

ATF: Bureau of Alcohol, Tobacco, Firearm and Explosives
CONAPO: Consejo Nacional de Población
INEGI: Instituto Nacional de Estadística y Geografía
NVDRS: National Violent Death Reporting System

## References

[CR1] Aburto JM, Beltran-Sanchez H, Garcia-Guerrero VM, Canudas-Romo V. Homicides in Mexico reversed life expectancy gains for men and slowed them for women, 2000–10. Health Aff. 2016;35(1):88–95. 10.1377/hlthaff.2015.0068 PMC5453309 26733705

[CR2] Beittel JS. Mexico: organized crime and drug trafficking organizations. Congressional research service; 2024. https://sgp.fas.org/crs/row/R41576.pdf. Accessed 23 Jan 2024.

[CR3] Bureau of Alcohol Tobacco Firearms and Explosives (ATF). Firearms Trace Data—2021; 2022. Available from: https://www.atf.gov/resource-center/firearms-trace-data-2021.

[CR4] Calderon LY, Heinle K, Kuckertz RE, Rodriguez Ferreira et al. Organized crime and violence in Mexico: 2020 special report. Justice in Mexico; 2024. https://justiceinmexico.org/wp-content/uploads/2020/07/OCVM-2020.pdf. Accessed 20 Jan 2024.

[CR5] Calderon LY, Heinle K, Kuckertz RE, Rodriguez Ferreira et al. Organized crime and violence in Mexico: 2021 special report. Justice in Mexico; 2024. https://justiceinmexico.org/wp-content/uploads/2021/10/OCVM-21.pdf. Accessed 20 Jan 2024.

[CR6] Castilla Peon MF, Rendon PL, Gonzalez-Garcia N. Homicides is the leading cause of death in children and adolescents in Mexico; 2023. Available at SSRN: https://papers.ssrn.com/sol3/papers.cfm?abstract_id=4601306.

[CR7] Centers for Disease Control and Prevention. Wide-ranging online data for epidemiologic research (WONDER); 2024. Available at: https://wonder.cdc.gov/. Accessed 30 April 2024. 10.1097/NMC.0000000000001201 42339962

[CR8] Centers for Disease Control and Prevention. National Violent Death Reporting System; 2024. https://www.cdc.gov/violenceprevention/datasources/nvdrs/index.html. Accessed 23 Jan 2024.

[CR9] Consejo Nacional de Población. Población a inicio de año, 1950–2070. Gobierno de Mexico. https://datos.gob.mx/busca/dataset/proyecciones-de-la-poblacion-de-mexico-y-de-las-entidades-federativas-2020-2070. Accessed 4 Jan 2024.

[CR10] Cunningham RM, Walton MA, Carter PM. The major causes of death in children and adolescents in the United States. N Engl J Med. 2018;379:2468–75. 30575483 10.1056/NEJMsr1804754 PMC6637963

[CR11] Dare AJ, Irving H, Guerrero-Lopez CM, Watson LK, et al. Geospatial, racial, and educational variation in firearm mortality in the US, Mexico, Brazil, and Colombia, 1990–2015: a comparative analysis of vital statistics data. Lancet Public Health. 2019;4(6):281–90. 10.1016/S2468-2667(19)30018-0 31126800

[CR12] Degli Esposti M, Coll CV, Murray J, Carter PM, Goldstick JE. The leading causes of death in children and adolescents in Brazil. Am J Prev Med. 2023;65:716–20. 36963471 10.1016/j.amepre.2023.03.015

[CR13] Goldstick JE, Cunningham RM, Carter PM. Current causes of death in children and adolescents in the United States. N Engl J Med. 2022;386:1955–6. 35443104 10.1056/NEJMc2201761 PMC10042524

[CR14] Hernandez Gress ES, Flegl M, Krtikj A, Boyes C. Femicide in Mexico: statistical evidence of an increasing trend. PLoS One. 2023;18(12):e0290165. 38134021 10.1371/journal.pone.0290165 PMC10745190

[CR15] Instituto Nacional de Estadística y Geografía. About the INEGI; 2023. https://en.www.inegi.org.mx/inegi/acercade.html. Accessed 4 Dec 2023.

[CR16] Instituto Nacional de Estadística y Geografía. Mortalidad. Subsistema de Información Demográfica y Social; 2023. https://www.inegi.org.mx/programas/mortalidad/#microdatos. Accessed 4 Dec 2023.

[CR17] Instituto Nacional de Estadística y Geografía. Población rural y urbana. Cuéntame de México; 2024. https://cuentame.inegi.org.mx/poblacion/rur_urb.aspx?tema=P#:~:text=%2C%201950%20%2D%202010.-,INEGI,de%20Poblaci%C3%B3n%20y%20Vivienda%202020.&text=En%201950%2C%20la%20cantidad%20de,ubica%20en%2021%20por%20ciento. Accessed 23 Jan 2024.

[CR18] Kivisto AJ, Lee Phalen P. Effects of risk-based Firearm Seizure laws in Connecticut and Indiana on suicide rates, 1981–2015. Psychiatr Serv. 2018;69(8):855–62. 29852823 10.1176/appi.ps.201700250

[CR19] McDougal TL, Shirk DA, Muggah R, et al. The way of the gun: estimating firearms trafficking across the US-Mexico border. J Econ Geogr. 2014;15(2):297–327. 10.1093/jeg/lbu021

[CR20] Perez Esparza D, Johnson SD, Gill P. Why did Mexico become a violent country? Secur J. 2020;33(2):179–209. 10.1057/s41284-019-00178-6

[CR21] Peters AW, Yorlets RR, Shrime MG, Alkire BC. Firearm-related fatalities in OECD countries, 2018–30: a value-of-lost-output analysis. Health Aff. 2020;39(11):1961–9. 10.1377/hlthaff.2019.01701 33136496

[CR22] Sun S, Cao C, Ge Y, et al. Analysis of firearm violence during the COVID-19 pandemic in the US. JAMA Netw Open. 2022;5(4):e229393–e229393. 35482307 10.1001/jamanetworkopen.2022.9393 PMC9051986

[CR23] Weigend Vargas E, Hans Z, Wiebe DJ, Goldstick JE. Firearm manufacturing and imports in the USA and their association to firearm homicides in Central America and the Caribbean, 1991–2019. Inj Prev. 2024. 10.1136/ip-2023-045055 PMC11291701 38302284

[CR24] Weigend Vargas E, Perez RC. Non-fatal gunshot injuries during criminal acts in Mexico, 2013–19. Inj Prev. 2022;28(3):238–42. 34887333 10.1136/injuryprev-2021-044411

